# Baltimore Almshouse Infirmary

**Published:** 1829-09

**Authors:** 


### BALTIMORE ALMSHOUSE INFIRMARY.

### Dr. Wright's *Reports of Cases of Erysipelas.*

The following cases are reported for the purpose of pointing out the very manifest controlling or modifying influence displayed by erysipelas over other forms of disease existing in the system at the time of its invasion.

Case I.—Hannah Kennard, forty-five years of age, full habit, and firm constitution, was admitted into the Baltimore Almshouse, June 7th, 1827, for treatment of a large inveterate ulcer of the leg, of four years' standing. Her general health was unbroken. On the third day after admission, this woman complained in the evening of being sick, feeling alternately chilly and feverous, with headach and nausea.—Ordered an emetic, to be followed by a full dose of calomel.

The following morning, the second of the disease, there was soreness, with swelling, heat, and inflammatory blush, over the integument of the forehead, face, and part of the scalp; the swelling greatest on the forehead. The pulse was frequent and full; febrile heat generally diffused over the body, tongue white, head painful. The woman attributed her indisposition (incorrectly, I think,) to having been washed in cold water when first admitted into the house. This was the first instance of erysipelas of the face and scalp occurring in the house, after the medical department of the institution had been placed in my charge. She was ordered small doses of calomel, nitre, and antimonial powder, at intervals of four hours, with an intervening dose of weak solution of tartar emetic, charged with spirit of nitre; drink, barley-water acidulated; diet, milk diluted, and toasted bread; free admission of air. The local affection was treated with solution of muriate of ammonia in distilled vinegar and water; the solution charged with a small quantity of the tincture of camphor, and applied cool, by means of linen cloths.

On the third morning, the face and scalp were swelled to great deformity; the palpebræ loaded and closed, the cheeks almost level with the spine of the nose, lips very thick and hard; the whole surface, forehead, and face, smooth and glistening from tension. The pulse more frequent than on the day before, still full, but less firm; the general heat increased; tongue brown, with white edges, and moist; head painful; mind somewhat unsteady.—The medicine was continued, with addition of camphor to the powders of calomel and nitre; and, on account of slow bowels, sulphate of magnesia was added to the draught. The local application was also continued, but, under an impression that it would be more soothing, and better relieve heat and tension, it was directed to be used pleasantly warm.

Afternoon.—Large and very much elevated vesications over the face; and which were directed to be carefully punctured, the effused fluid gently washed off with tepid water, and the warm ammonia lotion reapplied. On account of vigilance and restlessness, a full anodyne of Dover's powder was ordered for the night, after operation on the bowels.

Fourth morning.—The patient was a good deal prostrate, pulse contracted and quick, surface rather cool, tongue dry and brownish red, bowels lax; the face and scalp less swoln, surface of vesications dry and red. The patient was quieter than before, and rather disposed to somnolency, approaching stupor.—The treatment was now changed. Quinine was ordered, one grain in two hours, dissolved in acidulated aromatic water; with tincture of bark, spirit. mindereri every four hours, with ten drops of laudanum, to restrain the bowels. Local application continued as an occasional wash for the erysipelatous surface, which was after dusted with common starch. Diet rendered somewhat cordial, by a small addition of wine to the bread-tea, which had been previously used in a simple state.

Fifth day.—The energies of the system had rallied sensibly: irritation of the bowels allayed, pulse less frequent and more steady; the surface naturally warm, and the patient acknowledged improvement of feelings. The amendment continued.

On the sixth day, fever was absent; the patient sat up in bed, and took nourishment with sufficient appetite. Her complete recovery was established in a few days; the whole erysipelatous surface desquamated.

The ulcer of the leg, five or six inches every way in surface, which had remained in a foul, unhealthy condition for four years, in a few days after the fever of erysipelas subsided, filled up rapidly with granulations of good size and colour, and, under simple treatment, with rest, closed in with a firm smooth surface, in little more than a fortnight after recovery from her late illness.

Case II.—William Pine, aged about thirty, had been for many months in one of the cells of the Baltimore Almshouse, in a state of insanity. From what could be learned of his history prior to admission, he had been for some years in an unsettled state of mind, and had occasionally undergone medical treatment and moral restraint on that account. His understanding, at the time of his admission, seemed wholly obliterated, and he continued without after-evidence or interval of reason during the time stated, namely, for many months. His manner was generally silent and sullen, and, when he could be excited to any degree of effort, he spoke with rapid incoherence, his conversation running into a chaos of folly. The man's bodily health seemed little affected by his state of mind: he became somewhat emaciated from confinement, but his pulse was always calm and equal, and the temperature of body natural. He took food regularly, though without that voracity of appetite frequently attending insanity; and the excretions necessary to health were sufficiently performed. In respect to the latter, however, the patient had reverted to the state of childhood: the evacuations were passed without regard to time or circumstance.

The preceding statement is given to show the man's common state while in the house. On the 9th of June, Pine was attacked by chill, with high fever, flushed countenance, inflamed eyes, foul tongue, and the general evidences of great disorder. He was immediately removed from the cell to the infirmary, for the benefit of a purer and fresher atmosphere. On the day succeeding the chill, a deep florid hue overspread the face and scalp; swelling went on rapidly, attended by the smooth, glistening appearance characteristic of erysipelas, with effusion into the cellular structure of the eyelids, lips, &c. as described in the former case. The feverish tumult was much more marked than in the preceding instance, with severe headach, great heat of body, &c.

Already, in the first stage of this acute attack, Pine's intellect seemed to be rousing from its long torpor and habitual error. When questions were addressed to him now, there was an apparent act of deliberation before answering, and his replies, with occasional vacillation, were coherent and satisfactory. This relative saneness became more distinct and established as the disease advanced; and, on the third, fourth, and following days of its course, Pine's manner, replies, &c. were orderly and rational, no longer betraying any tendency to alienation. The local inflammation and constitutional disorder were very severe in this case for some days. The former resulted in early and extensive vesication, terminating ultimately in general desquamation of the cuticle of the face. The fever remitted on the fourth day, and subsided altogether about the sixth, (its nearly uniform period of cessation in almost all the cases,) leaving the patient much debilitated.

The treatment was conducted by moderate purging with calomel, followed by Epsom salts, with minute addition of tartrate of antimony. The subcarbonate of potash, with wine of antimony or ipecacuanha, was afterwards given generally through the course of fever, in doses and at intervals regulated by its effect on the stomach, pulse, and skin. The patient's drink, diet, and general regimen was the same as in the case before reported; and the local application used in the other case was employed in this also.

The intelligence which had been revived in Pine's case with the development of erysipelas, continued during his convalescence, and he left the institution without having betrayed indications of lapsing into his former obliquity of mind. This man abused the privilege granted to convalescents of walking in the yard for air and exercise, and eloped from the Almshouse ten days after recovery from erysipelas. His state since then is not known to me.—*American Journal of Med. Sciences.*

---

*Case of Hydrothorax, partial Ascites, and great Infiltration of the lower Extremities.* By Thomas H. Wright, m.d.

The following case supplies a satisfactory explanation of one of the causes of sudden death, ensuing to hydrothorax, after the signs proper to that form of dropsy were no longer perceptible, and when the case seemed to have put on the character of decided convalescence.

Charles O'Neal, labourer, aged forty-seven, was admitted into the Baltimore Almshouse Infirmary, June 20th, 1827. The symptoms of thoracic and general dropsy commenced four months antecedent to admission, and were consequent on cold, fatigue, and long exposure to wet, in the laborious occupation of ditching, in which he had been employed the greater part of the preceding winter. The difficulty of breathing was now great, with inability to lie down; cough inconsiderable, slight fever, no acute pain, bowels torpid, and urine scanty.

This patient was put on the use of a bitter cathartic infusion, (gentian, senna, supertartrate of potash, and aromatics,) which kept the bowels freely soluble, and increased the secretion of urine, but without sensible alleviation of the symptoms of hydrothorax. The breathing continued anxious, difficult, and crepitating; and the inability to lie down for more than a few seconds remained as at first. The infusion was continued, with the addition of a diuretic compound, (tinct. digitalis, ol. terebinth., and spt. nit.) exhibited along with the cathartic draught. The purgative and diuretic effects of the combination were very considerable, and both the ascites and anasarca were very greatly reduced. No favorable impression, however, was made on the hydro-thoracic affection, and the agents hitherto employed were laid aside. The after-treatment consisted of a liberal use of solution of cream of tartar, sweetened with molasses, in a draught of which was occasionally exhibited the following diuretic compound: Fecula of Elaterium two grains, Tinct. Scill. and Oxymel Colchici, each half an ounce, in four ounces of solution of Acetate of Potash; dose, two to three drachms, pro re nata. The salutary operation of those agents soon became manifest, and was very explicit. In five or six days after adopting the latter course, the respiration became easy, measured, deep, and inaudible, having lost all crepitation. The breast was also now more distinctly resonant to percussion, and recumbency was practicable without inconvenience.

It was about the 1st of August, six weeks after admission, that the propitious circumstances just recorded occurred, and gave such indications of amendment as seemed to remove all cause of apprehension for the present. On entering the infirmary, 6th of August, it gave me great pleasure to notice this patient, dressed and sitting on his bed, with a countenance strongly expressive of the ease and comfort of his present feelings, contrasted with his distress for many weeks preceding. His remarks were full of confidence in his agreeable sensations, and exultation at the prospect of prompt and perfect recovery. Two hours after leaving the infirmary, I was called to return there in haste, on information that O'Neal had been seized with a fit. In a few minutes from his attack I was at his bedside, and he was without a sign of life.

I was at a loss to account for the catastrophe in this case, and my conjectures hesitated between the presumption of lesion in the brain, or sudden extinction of the heart's action, from concentrated dropsy of the pericardium.

*Dissection, a few hours after death.*—The head was first examined. The dura mater presented nothing uncommon, except a degree of humidity greater than occurs in the natural state; the proper tunics of the brain were also overspread by a very sensible quantity of thin serous fluid, constituting a remarkable dampness of the surface rather than any positive collection of fluid, though the amount much exceeded the natural halitus with which the parts are bedewed. There was some degree of engorgement of the superficial sinuses of the brain, but no mark of inflammation of the tunics. Cutting away the medullary substance cautiously down to the lateral ventricles, they were found very much enlarged, as if recently greatly distended, but were at this time quite empty. On prosecuting the dissection below the level of the tentorium, the fourth ventricle, the head of the medulla spinalis, and whole base of the brain, were inundated by water, which, when the head was depressed, flowed out to the amount of five or six ounces.

On referring now to the enlarged and empty state of the lateral ventricles, it seemed apparent that the fluid found upon the base of the brain had been first accumulated in the lateral ventricles; had remained there for an uncertain time without serious consequences; and at last, by forcing the valvular defence, and the pulpy closure of the passage leading to the fourth ventricle, had dropped suddenly upon that cavity, and by rupture of the ventricle (at its thin, inferior surface,) was effused upon the medulla oblongata, and into the spinal canal. Pressure to a great degree thus suddenly made on the origin of the most important nerves of organic life, seems fully adequate to explain the total extinction of sensorial function, with the nearly instant death that ensued in the case of this patient. In the thorax, the sacs of the pleuræ and the pericardium, there was very little effusion; nor was there any obvious morbid state of the heart itself, or about the origin of the great vessels.

The circumstances attending this case, its mode of termination, and the facts disclosed by examination tending to render that mode of termination intelligible, all seem to point to a certain inference in the present, and in relation to similar cases. They lead to the conclusion that, in dropsical disease, while effusion is going on in the serous and cellular textures of the great cavities and general body, a similar result may obtain in the delicate ventricular tissues of the brain, and especially of the greater or lateral ventricles.* It seems probable, also, that this partial hydrocephalic state may remain inoperative for a time, perhaps in some instances a long time, until, from quantity and gravitation, or some shock causing its irruption, it may suddenly inundate the base of the brain, and produce instant death, by invading the seat and suppressing the function of the most important nerves. The event in contemplation may occur at some stage of progressive dropsy, or may ensue at various intervals subsequent to apparent removal of the general hydropic state; for it is probable, from many facts and analogies, that hydrocephalic accumulations are seldom removed or diminished by absorption.†—*Ibid.*

---

*Anasarca and Ascites. Fatal and Sudden Termination.*
By Dr. Wright.

William Tarring, aged twenty-six, was admitted into the Baltimore Almshouse, September 17th, 1827. This was an aggravated case of general dropsy, uncomplicated, however, by hydrothorax. Abdomen extremely prominent and tense; upper and lower extremities greatly œdematous; countenance eminently leucophlegmatic; great debility; breathing short, but clear,

* It is the suggestion of some eminent anatomists (the Bell's,) that the plexus choroides is almost entirely a web of vessels destined to the secretion of a fine serum or halitus. If this opinion be well founded, it may explain why great serous accumulation is more generally found occupying the lateral ventricles than the other cavities of the brain, as only a very faint appearance of choroid plexus can be discovered in the other ventricles.

† A surmise of absorption has been founded on some examinations of the head, in which previous hydrocephalic collection was inferred from certain indications in the state of the membranes and ventricles, though there was no longer sensible effusion. Yet the long period, sometimes years, for which the water of hydrocephalus is known to have remained unabsorbed, taken in connexion with the fact that cases attended by unequivocal signs of matured hydrocephalus are very generally fatal sooner or later, places the presumption of absorption to an extent sufficient to relieve dropsy of the brain, on ground of doubtful authority and difficult admission.

without mark of bronchial effusion; no cough, and absence of fever. The dropsical affection had existed some weeks prior to admission, and was consequent on severe remittent fever, contracted in August, while working on the Harrisburg canal.

The excessive tension of the abdomen in this case prevented any satisfactory examination, by taxis, of the great viscera of the belly, the liver, spleen, &c.; but, from the patient's report of having been in good health previous to the attack of remittent fever, on which the dropsy was consequent, and from the absence of any representation of serious hepatic derangement in the complexion, the tongue, excretions, &c. of the patient, the case was presumed to be free of any actual organic impairment. Some degree of primary visceral congestion, high and continued tumult and effort of vascular action, with more or less irritation and disorder of the gastro-hepatic functions, and proportionate exhaustion ensuing to those joint causes of disturbance, seemed to have led on rapidly to hydropic effusion, with the disabled and oppressed state of the system which the case presented on admission.

The usual remedies of dropsy (omitting in great part mercurial combination,) were employed in the case as freely as circumstances would permit. An infusion of gentian, senna, supertartrate of potash, and aromatics, was given liberally, with powders of squill, nitre, and digitalis; Dover's anodyne being added to the powder exhibited at night. The patient was also directed the free use of barley-water, acidulated with cream of tartar, and rendered moderately cordial by gin. Under this treatment both the bowels and kidneys performed their appropriate functions with as much activity as was desirable; the tension of the belly was gradually taken off, and the overloaded cellular tissue became relieved. The evacuation of the dropsical effusion went on slowly but constantly, and, by the careful regulation of the medicines, with due alimentary support, the patient was free of oppression, and somewhat recruited in strength, by the end of the fifth week after admission. This man became able to leave his bed, and walk the infirmary hall every day for some hours, eating with appetite his whole allowance of food.

During this promising state of convalescence, Tarring was attacked, in a season of wet weather, late in October, by quotidian intermittent fever, for which bark and aromatics were given freely, and the paroxysms were interrupted after the third period. But the patient was a good deal worsted by the attack, was confined to bed by debility, and, in a few days subsequent to arrest of the intermittent paroxysms, dropsical effusion began to display itself in the extremities. In a week after he was as much affected by ascites and anasarca as when first admitted.

The remedies successful in the first instance were tried again, more sparingly, however, in quantity; but their good effect was not so considerable as formerly, the action of the absorbents, of the kidneys, and of the bowels, being much less efficient; and, after a longer perseverance in the medicines than had been necessary to effect improvement in the first instance, the amount of effusion remained undiminished, while the energies of the constitution had gradually declined.

The medicinal agents were then changed: good Lima bark, with an aromatic, was ordered, and the patient directed a liberal use of the cream of tartar punch, with the addition, three or four times in twenty-four hours, of two drachms of a diuretic combination before noticed: namely, fecula of elaterium four grains, in four ounces of solution of acetate of potash, adding tinct. of squill and oxymel of colchicum.

This plan of treatment, with light cordial nourishment as freely as could be taken, gave a better aspect to the case. The urine augmented very much, the alvine evacuations became copious and watery, the tension every where relaxed gradually, and the forces of the circulation rallied to the natural tone as the oppression was taken off.

Early in December the signs of dropsy were a second time dissipated, and the youth of the patient, in connexion with a naturally good constitution, encouraged the expectation of permanent convalescence.

Nothing occurred to defeat the presumption of perfect recovery in this case until the 20th of December. The man had been about all day, and on that evening had eaten a hearty supper, and seemed as well and comfortable as for some time before. An hour after supper the nurse of the ward was called to Tarring, by a patient in an adjacent bed, who observed him fall suddenly into a state of unusual agitation and disorder. She found him with limbs rigid; oppressed, stertorous respiration; and in a state of insensibility from which he could not be roused. He expired in a few minutes.

From circumstances which rendered autopsic examination inconvenient at the time, the direct cause of sudden death in this case was not ascertained. But, from the marked analogy in the manner of Tarring's death, with previous fatal instances under like general circumstances, where serous extravasation was found to have occurred in the basis cerebri and medulla oblongata, the issue of his case was referred to a similar cause. The period of life in this case was much against the probability of organic degenerescence of structure about the organ of circulation; and the presumption of serous, cardiac, or pulmonic infiltrations, was equally opposed by the non-existence of even the slightest hydro-thoracic symptoms at any period of the dropsical affection.—*Ibid.*

*Case of extensive Ulceration of the Leg, healing rapidly in an unexpected manner. Death. Effusion into the Ventricles of the Brain.* By Dr. Wright.

Thomas Coursey, a black man, aged seventy-four, of very large frame of body, and apparently great original vigor of constitution, was admitted into the Baltimore Almshouse, in September 1827. This man was in good general health when admitted, yet was disabled from work partly by age, but more from disease of the left inferior extremity. The motion of the left knee-joint was entirely lost, in consequence of severe contusion of the part received thirty years before. From the man's account, arthritis to a great degree had ensued to the injury in the first instance, followed by profuse suppuration, great distention, and ultimate relaxation of the synovial membrane, articular and capsular ligaments, and finally spontaneous subluxation, outward and backward, of the head of the tibia; which, becoming permanent, caused a very curious and rare species of deformity.

Besides the distortion and anchylosis of the knee-joint, the leg was overspread by a large and deep ulcer of the chronic indolent class, which had commenced simultaneously with, or very soon after, the injury of the knee, thirty years past, and had never healed. The surface of ulceration was altogether, at the patient's admission into the infirmary, about ten inches in extent, suppurating profusely; and, from its seat, appearance, and duration, had probably long since involved a diseased state of the periosteum and bone.

The man's general health being good, nothing further was directed in his case than rest, and such local treatment as was indicated by the state of the ulcer, namely, cleanliness, and dressings fitted to maintain, as far as possible, a healthy state of the suppurating surface. From the man's age, the character and duration of the ulcer, its complete closure or cicatrization was not contemplated or deemed probable.

This man remained well for three months succeeding his admission, evincing no disposition to any form of serious or constitutional disease; his spirits, appetite, and personal appearance were all perfectly well maintained. The ulcer of the leg during this time had undergone, as was expected, scarcely any sensible change, other than the clean appearance and absence of irritation which frequent and regular dressing, with continued rest, would almost necessarily produce: its dimensions were but little diminished. Late in December, however, a sudden and remarkable alteration was displayed in its character. It commenced a course of rapid healing, and, in ten days from the time the change was first noticed, the whole original space of the ulcer was covered in,* and

* A man named Lafito, who had been long a dresser in the house, and formerly (as he represents) in the same capacity in the Philadelphia Almshouse, remarked, while exhibiting the great change in the state of Coursey's leg, cicatrized very smoothly, leaving only a central point a few lines broad, which furnished a partial suppuration.

No obvious difference in the patient's general health attended this sudden healing of the ulcer, but a very prominent change was manifested in the man's temper and moral habits. He became peevish, quarrelsome, and indocile in his conduct, averse to keep in his place or put up with his accommodations in the ward, and very impatient of admonition or control. His language and manners betrayed nothing of derangement of intellect, properly so called: yet his fretful, unquiet state, and improper manner of speaking, rendered it at last necessary to remove him from the ward to one of the cells, where he was kept alone, but without any personal constraint. This was done in the second week after the healing of the ulcer, and, on the day following his removal to the cell, Coursey was visited by the superintendent of the institution, who found him apparently well as usual, but, as before, very much disposed to rude and passionate complaints, which he indulged to a great degree on the present occasion. In a few minutes after the visit of the superintendent, the keeper of the cells came to report that Coursey had fallen down in a fit, and died instantly.

*Examination, four hours after death.*—The dura mater exhibited nothing uncommon, except an unusual blanched appearance, and was without marks of inflammation or of vascular engorgement. The proper tunics of the brain presented a natural condition, or were in no other manner altered in character than by excess of humidity, and the same blanched or soddened appearance observed about the dura mater.* This humid and bloodless appearance was obvious in the successive portions of the medullary matter, removed in penetrating by sections down to the lateral ventricles. When those cavities were arrived at, and carefully opened, they presented the most extraordinary degree of enlargement I have ever witnessed. The ventricles were penetrated at the posterior horn, and were found empty; but, on enlarging the incision, and lifting the thin covering which had been left them in removing the cerebral mass above, the ventricles presented rather the appearance of caverns than cavities in the brain, seeming capable of receiving a body of considerable size, (as a small orange or lemon,) and of space sufficient to contain several ounces of fluid. On being fully exposed, the delicate tunic reflected around the ventricles was of highly florid colour throughout, from minute vascular engorge-

"that the man had not long to live:" an idea of danger from the sudden healing of old ulcers not always realized, yet far from being altogether a vulgar notion or groundless prejudice.

* The convolutions of the cerebrum were much deeper, and more readily and widely separable from each other on this subject than I had ever noticed them in any other instance. In all those fissures, and to a great depth between the convolutions, might be seen bundles of vessels running in straight lines, or stretched across the interspaces, and all those vascular fasciculi, (the deep arteries and veins of the pia mater,) of high colour, and very much engorged.

ment, while the plexus choroides was a good deal wasted, and altogether colourless, not easily distinguishable, and much resembling a gelatinous mass. The valvula cerebri (of Vieussens) could not, from distention of parts, be recognised; the aqueduct of Sylvius was very much dilated, and, from its commencement down into the fourth ventricle, was filled with water; as was also the fourth ventricle, the whole space around the base of the brain and head of the spinal marrow; the same fluid gravitating freely into the vertebral canal. The quantity of water poured out of the inferior cavities and space of the brain, and of the upper vertebral cavity, exceeded six ounces; its colour was slightly red, and it was somewhat viscous or albuminous to the feel when rubbed between the fingers.*

In this case, circumstances warrant the presumption that the great quantity of water found occupying all the lower cavities and space of the head was originally produced in, and for some time confined to, the lateral ventricles; from whence it descended, probably suddenly, into the fourth ventricle, (which was ruptured inferiorly,) and thus became effused and free as it was found on dissection. The cavern-like enlargement of the lateral ventricles indicated great distention by previous accumulation of fluid.

The phenomena arising towards the close, and concurring in the termination of this case, viewed in connexion with the direct cause of that termination revealed by dissection, bear a seeming intimate relation to two points of physiology. They tend to illustrate the liability and mode of sudden death from large collections of water in the upper cavities of the brain, and serve in the present case forcibly to call to mind the ancient and much-controverted doctrine of the metastatic attribute of disease. A doctrine fruitful of medico-scholastic dispute, which, after various fortune and a period of protracted dormancy, has been revived under the light of better science, and become a good deal the favorite of modern pathology.† Can we allow ourselves the inference in the case before us, that the sudden healing of an extensive

* The commissura mollis, so seldom to be found that its existence has been questioned by some eminent anatomists, was very distinct in this case. It was of pale, yellowish gray colour, semi-translucent, about the size of a writing quill, standing as a bridle across the thalam. nerv. opt. under the fornix. Mr. Bell is probably right in saying that the commissura mollis is best seen in hydrocephalic brains. I have not discovered it plainly in any other.

† In substituting the metastasis of morbid actions for the original notion of a translation of morbific matter, or the migration of disease in its limited and literal sense, modern pathology has taken a ground so well sustained by observation and facts, as to be perhaps impregnable. That on the sudden suspension of morbid actions in one seat, irritation and its consequences may be simultaneously developed in some other part, the latter holding a relation of dependence or substitution to the former, seems entitled to rank as an axiom of medicine.

ulcer of thirty years' standing, was concerned in the change represented to have taken place at that time in the patient's conduct and manners, and that the moral effect in question resulted from physical irritation devolved on the encephalon? Can we further presume, on legitimate metastatic principles, that the sudden suppression of habitual suppurative action over an extensive surface on the leg, might give occasion to that excitement to serous exhalation into the cavities of the brain, the product of which was so prominently obvious in this patient's case.

Regarding it as a curious fact, that a rapid and apparently sound closure of an extensive ulcer of thirty years' continuance should have occurred, where I felt assured that the subjacent bone had suffered degeneration, probably in a great degree, I caused a portion of tibia to be cut out, and subjected to maceration for a sufficient time to free the bone of all soft matter. When this was effected, and the bone dried, the seat and extent of the ulcer were found very clearly and strongly defined on the tibia. There existed a very peculiar change, which the surface of bone, corresponding to the ulcer, had undergone, which was not caries or necrosis in any of their forms: the bone was firm and solid over its whole extent, but a singular state of granular and scabrous roughness occupied the space of bone answering to the seat of the ulcer. There existed, in fact, for some inches on the face of the tibia, a dense crop of exostoses of various form and dimensions, some of the miliary character, and small size; others, orbicular or pisiform, of considerable magnitude, and many scaly or laminated, with broad surface, raised on a sort of footstalk, and were thus button shaped, or much resembling small fungi of the mushroom species. All those productions were white and solid, (not easily broken off,) and were apparently complete specimens of ossification. The possibility of the formation by gradual interstitial waste of bony substance was taken into consideration; but there were no evidences of such interstitial waste having occurred, there were no marks of softening or caries upon or around the surface affected; and the circumference of the bone, taken between the eminences of the affected surface, was equal to the shaft of the bone above or below the limits of disease.—*Ibid.*

